# Test-Time Augmentation for Cross-Domain Leukocyte Classification via OOD Filtering and Self-Ensembling

**DOI:** 10.3390/jimaging11090295

**Published:** 2025-08-28

**Authors:** Lorenzo Putzu, Andrea Loddo, Cecilia Di Ruberto

**Affiliations:** 1 Department of Electrical and Electronic Engineering, University of Cagliari, 09123 Cagliari, Italy; 2 Department of Mathematics and Computer Science, University of Cagliari, 09124 Cagliari, Italy; cecilia.dir@unica.it

**Keywords:** domain shift, domain generalisation, test-time augmentation, self-ensemble, late fusion, weighted voting, leukocyte classification

## Abstract

Domain shift poses a major challenge in many Machine Learning applications due to variations in data acquisition protocols, particularly in the medical field. Test-time augmentation (TTA) can solve the domain shift issue and improve robustness by aggregating predictions from multiple augmented versions of the same input. However, TTA may inadvertently generate unrealistic or Out-of-Distribution (OOD) samples that negatively affect prediction quality. In this work, we introduce a filtering procedure that removes from the TTA images all the OOD samples whose representations lie far from the training data distribution. Moreover, all the retained TTA images are weighted inversely to their distance from the training data. The final prediction is provided by a Self-Ensemble with Confidence, which is a lightweight ensemble strategy that fuses predictions from the original and retained TTA samples using a weighted soft voting scheme, without requiring multiple models or retraining. This method is model-agnostic and can be integrated with any deep learning architecture, making it broadly applicable across various domains. Experiments on cross-domain leukocyte classification benchmarks demonstrate that our method consistently improves over standard TTA and Baseline inference, particularly when strong domain shifts are present. Ablation studies and statistical tests confirm the effectiveness and significance of each component.

## 1. Introduction

White blood cells (WBCs), medically known as leukocytes, are essential components of the immune system. They protect the body against microbial invasions and pathogens. Classifying WBCs is crucial in healthcare as it provides important information for diagnosing diseases and guiding treatment decisions [1]. WBCs are primarily categorised into five sub-types: neutrophils, eosinophils, basophils, lymphocytes, and monocytes, each performing specific functions in the immune system. Maintaining a normal WBC count is essential for overall health and well-being [1]. Conventional methods for classifying WBCs, such as manual microscopy, often suffer from long processing times and are prone to human error. In recent years, Machine Learning (ML), especially deep learning (DL), has emerged as a promising and transformative approach for WBC categorisation, offering the potential to enhance the precision of WBC classification while mitigating the risk associated with human fallibility [1,2].

Based on extensive training datasets comprising meticulously labelled WBC images, DL algorithms acquire the ability to discern and categorise distinct leukocyte varieties autonomously. However, the automation of this procedure faces challenges, beginning with clinical factors such as the diverse shapes and structures of cells and variations in protocols across different hospitals, including differences in equipment, lighting, and staining procedures. These variations can result in a domain shift that significantly impacts the performance of DL models trained solely on In-Distribution (ID) data from a single site when applied to Out-of-Distribution (OOD) images belonging to different sites. To address the domain shift issue, existing solutions rely on domain adaptation (DA) [3,4] or domain generalisation (DG) [5] methods. These approaches aim to improve the model’s robustness in the target domain by utilising data, labelled (common DA) or unlabelled (Unsupervised DA—UDA), from the target domain or by extracting domain-invariant features from multiple domains. Due to privacy restrictions, collecting and aggregating data from the target domain or multiple domains simultaneously is often unfeasible in real-world applications.

In this work, we introduce a straightforward solution that utilises data augmentation at testing time, which is known as test-time augmentation (TTA). Data augmentation is typically employed during training to produce variations of the original sample images, intended to develop DL models more resilient to image variations, thus enhancing their ability to generalise to new samples [6,7]. Recently, instead, data augmentation has also been used at testing time as a powerful heuristic that takes advantage of data augmentation during testing to produce averaged output [8]. Existing methods of TTA typically apply a predefined set of transformations to the input and average the model’s predictions, without assessing the reliability or relevance of each augmented sample [9,10,11,12]. This naive approach may incorporate misleading predictions from low-quality or OOD samples, especially in cross-domain scenarios, ultimately limiting its effectiveness [13].

In this work, to mitigate such limitations, we introduce a filtering mechanism based on OOD detection in the deep feature space, aimed at retaining only informative TTA samples during inference. Our approach is lightweight, model-agnostic, and easily integrable into standard TTA pipelines, particularly in medical imaging tasks where cross-domain generalisation is a major challenge. The DL models used are trained in a supervised fashion, without any search for optimal augmentation policies or task-specific loss functions. At inference time, a fixed set of transformations is applied to generate augmented test samples. This design choice ensures reproducibility and fairness across datasets and architectures and reflects the fact that in the unsupervised test-time scenario considered here (see Section 3.1), no access to target-domain data is available for tuning augmentation strategies. The augmented samples are then filtered using a k-Nearest Neighbour (k-NN) strategy operating on multi-layer deep features, discarding transformations deemed Out-of-Distribution. The retained samples are ensembled with the original input through a weighted soft voting scheme [14] within a self-ensembling framework, improving robustness without requiring access to target-domain data or training multiple models. Our main contributions are as follows:

- We propose a TTA procedure, in order to exploit the knowledge already learnt by the DL models during training, even during inference;
- We propose an OOD filtering procedure that exploits multi-layer deep features and the Euclidean distance to filter out the generated samples that are too far from the ID samples;
- We propose a fusion method that improves the classification performance by fusing the original ID data with generated TTA samples without the need for external data;
- We create a Self-Ensemble classifier that leverages a single DL model to provide a prediction based on the information of both ID and TTA through weighted soft voting;
- We provide a model-agnostic solution that can be used with any DL architecture for WBC classification and potentially can be used for every image classification task.

To demonstrate the effectiveness of our solution, we conducted a comprehensive cross-data evaluation on four benchmark datasets. We exploited different DL architectures, including Convolutional Neural Networks (CNNs) and vision transformers (ViTs), demonstrating that the proposed solution effectively addresses the domain shift in WBC classification. The remaining sections of the manuscript are structured as follows. Section 2 outlines relevant previous research on WBC analysis and solutions against domain shift. Section 3 presents the considered reference scenario and the proposed solution. Section 4 describes the materials, methods, and settings used in our evaluation and the obtained results. Lastly, Section 5 provides conclusions and discusses potential future research.

## 2. Related Work

The advent of Computer-Aided Diagnosis (CAD) systems has ushered in a new era, promising to revolutionise the process of cytological evaluation of WBCs in blood or bone marrow smears by automating and enhancing diagnostic accuracy and efficiency [15,16,17,18,19]. Existing CAD systems for WBC analysis encompass a spectrum of tasks, ranging from simple cell counting and passing through cell classification to the detection and classification of diseases. For the sake of brevity, in the following, we focused on existing methods for WBC analysis (see Section 2.1), on existing solutions against domain shift in the same task (see Section 2.2), and on existing approaches based on test-time augmentation.

### 2.1. WBC Analysis

Most of the existing methods for WBC analysis are devoted to the classification of the five main WBC sub-types [15,20,21,22,23,24] or on the identification of particular cell precursors identifying specific leukaemia conditions, such as acute lymphoblastic leukaemia (ALL) or acute myeloid leukaemia (AML) [19,25,26].

The first methods on WBC classification exploited generic DL architectures [15], while the main advancements on the field are brought by the creation of novel architectures, either CNNs [22,27,28,29,30] or ViTs [24,31,32], ensemble strategies [33], or hybrid methods [21,23,34,35], potentially combining feature selection techniques [36].

A significant advancement in the field is exemplified by the development of BloodCaps by Long et al., a capsule-based model specifically designed for the accurate classification of various blood cell types in peripheral blood images. BloodCaps surpassed the performance of traditional CNNs, such as AlexNet, VGG-16, ResNet-18, and InceptionV3, underscoring the potential of novel architectural paradigms to improve classification techniques [27].

Furthermore, researchers have explored hybrid approaches, combining image processing and DL methods to achieve outstanding classification accuracy. For instance, Şengür et al. demonstrated the efficacy of such an approach in Whole Blood Cell Count (WBCC), underscoring the synergy between traditional and modern techniques [37], while Huang et al. developed a WBC classification framework by combining modulated Gabor wavelet kernels and deep CNN kernels, enabling the network to learn representative features at different frequencies and orientations [18].

Other authors focused not only on performance but also on computational costs. For such a purpose, Firat et al. introduced a novel multi-branch lightweight CNN architecture featuring three parallel branches: the Inception module, depthwise squeeze-and-excitation block (DSEB), and pyramid pooling module (PPM). The Inception module enhances efficiency and classification accuracy by conducting simultaneous convolutions at multiple scales. The DSEB selectively identifies informative features while eliminating redundant ones, requiring minimal computational resources. Additionally, the PPM captures multi-scale contextual information from input images through feature pooling at various scales [22]. Methods aimed at training with limited data, such as few-shot learning approaches [28], have received limited attention due to the prevalence of large datasets in this domain, akin to the ones utilised in this study.

### 2.2. Overcoming Domain Shift

Regardless of the type of supervision used, novel architectural proposals might encounter common challenges in the event of variations in lighting and stain colour or blurred boundaries introduced by different staining protocols across different hospitals. The resulting domain shift can severely affect the performance of models trained with data from a single site only and render established approaches ineffective at a specific site, requiring re-annotation and retraining of the models. Exposing optimisation to domain shifts may be a solution to aligning different domains in real-world data, e.g., with DA [3,4] or DG [5] methods.

Many methods for medical image analysis focus on aligning the feature distributions of the source and target domains to minimise the domain shift [3,38] using target reconstruction [39], adversarial learning [40,41], divergence minimisation [42], or domain randomisation [43].

A few studies have addressed domain shift in WBC classification [3,4,44,45]. In particular, a target-independent DA technique using CNNs was proposed in [3]. Such an approach comprises two stages: feature extraction, where a pretrained CNN extracts source domain features, and domain adaptation, where a domain classifier aligns source and target domain feature distributions using domain adversarial loss. A continual learning approach was in [44] for class incremental and domain incremental scenarios in WBC classification. Based on a CNN, this method is trained on a source domain and fine-tuned on a target domain, ensuring adaptability to new domains and classes. It involves selecting the most confident and challenging samples identified through uncertainty estimation of the model.

Unsupervised feature extraction for single-blood cell image classification was used in [4]. This method learns domain-invariant features from unlabelled images of the source domain using a deep convolutional autoencoder and then uses them to classify images of the target domain. Another study [45] addressed various challenges related to this task, such as dealing with imbalanced data, missing classes, and domain shifts stemming from differences in class distributions across datasets from different hospitals. To tackle these challenges, the researchers proposed a label-distribution-aware margin loss to handle data imbalance and a domain-adaptive regularisation method to address domain shifts.

Although DA and DG offer promising solutions, it is crucial to recognise that (1) access to the target domain or knowledge of all domains involved is typically not feasible in real-world applications and (2) sharing medical imaging data across multiple centres is challenging due to privacy protection regulations, such as the Health Insurance Portability and Accountability Act [46] and General Data Protection Regulation [47]. Consequently, new approaches are needed to create accurate models without accessing private patient information or transmitting raw data, as presented in other case study fields [48,49].

### 2.3. Test-Time Augmentation

TTA is a widely adopted strategy in computer vision for improving the robustness and generalisation of DL models during inference. By generating and aggregating predictions over multiple augmented versions of the same input image, TTA aims to reduce overfitting and sensitivity to input perturbations. In the medical imaging domain, where data variability is high due to differences in acquisition protocols, equipment, and institutions, TTA has shown promise in stabilising predictions and improving accuracy [9,10].

Several recent works have leveraged TTA in diverse application domains. For instance, in traffic sign detection under adverse conditions, TTA with simple geometric transformations such as scaling and flipping has been used alongside ensemble models to increase detection accuracy on corrupted or occluded signs [11]. In the context of skin lesion classification, TTA has been applied to multiple CNN architectures to marginally improve balanced accuracy, demonstrating its usefulness even in high-performance models [10]. Interpretability tools like t-SNE and Grad-CAM have also been coupled with TTA to build physician trust in AI systems.

More advanced uses of TTA have been explored in adaptation settings. For example, feature-level augmentations and perturbations have been proposed to maximise the use of limited test data in test-time adaptation scenarios, improving cross-domain performance without accessing source data [13]. In crop disease detection, isolated test-time adaptation techniques combined with strong and weak augmenters have been designed to address domain shift in agricultural settings [12].

Despite its advantages, TTA is not without limitations. As shown in several works, naive application of TTA can introduce unrealistic or OOD inputs, particularly in cross-domain settings where the augmented views may not reflect the true distribution of the target domain. This limitation has motivated hybrid approaches that combine TTA with adaptation strategies or filtering mechanisms to ensure that only reliable augmentations contribute to the final prediction [13].

In this work, we follow this line of research by introducing a principled filtering mechanism based on the concept of OOD detection in the deep feature space, aiming to retain only informative TTA samples during inference. Our approach is designed to be lightweight, model-agnostic, and easily integrable into standard TTA pipelines, especially in medical scenarios where cross-domain generalisation is critical.

## 3. Proposed Method

This section provides an overview of the proposed method. It begins with a description of the considered scenario and issues in Section 3.1, followed by the detailed description of the proposed methodology (Section 3.2).

### 3.1. Reference Scenario

As the existing literature indicates (see Section 2.1), prevailing methods for WBC classification typically rely on a conventional training and testing setup using benchmark datasets. In this conventional approach, DL models are trained on a singular set of fully labelled images and subsequently evaluated on a distinct partition of the same dataset, a setting known as the same-dataset evaluation setting. This method simulates a scenario wherein images are collected and labelled within a single haematological research centre. While this approach may yield exemplary performance on benchmark datasets [19], it remains somewhat detached from reality. Indeed, in practical application scenarios, acquiring labelled WBC images from the *target* haematological research centres, those that will employ the WBC classification system, is often unfeasible. Consequently, a model trained on a different (*source*) dataset must be utilised in a cross-dataset setting. However, despite their numerous variations, benchmark datasets are subject to significant dataset biases, similar to other computer vision tasks [50], thereby significantly impacting cross-dataset performance [19].

Data augmentation on the feature space is an approach to reducing such biases. It means that at training time, different versions of the original training images are created from an augmentation space that consists of (1) a set of augmentations *A* and (2) a set of possible strengths *s* (e.g., the rotation operation and the number of degrees, respectively). Including such augmented samples in the training phase is beneficial to reduce overfitting and promote generalisation by defining a decision function that accounts for these variations [6,7]. Despite their improved performance, using the samples of the source domain for feature space data augmentation may limit the search space and can hardly cover the feature space from the unseen target domains.

An alternative is to use data augmentation at testing time, which exploits a similar concept. Indeed, if a DL model *C* is trained on real and augmented data with a specific augmentation $A_{i}$, then $A_{i}$ produces images that lie within the domain of the class and is beneficial even at testing time. Nevertheless, even if this assumption is almost always true for samples that belong to a target dataset that coincides with the source dataset, it is often false for samples that belong to datasets that differ from the source one. Indeed, even in this case, the samples from the target domain hardly fall into the feature space of the source domain. For this reason, a procedure to filter out such samples is fundamental.

### 3.2. Methodology

**Figure 1:**
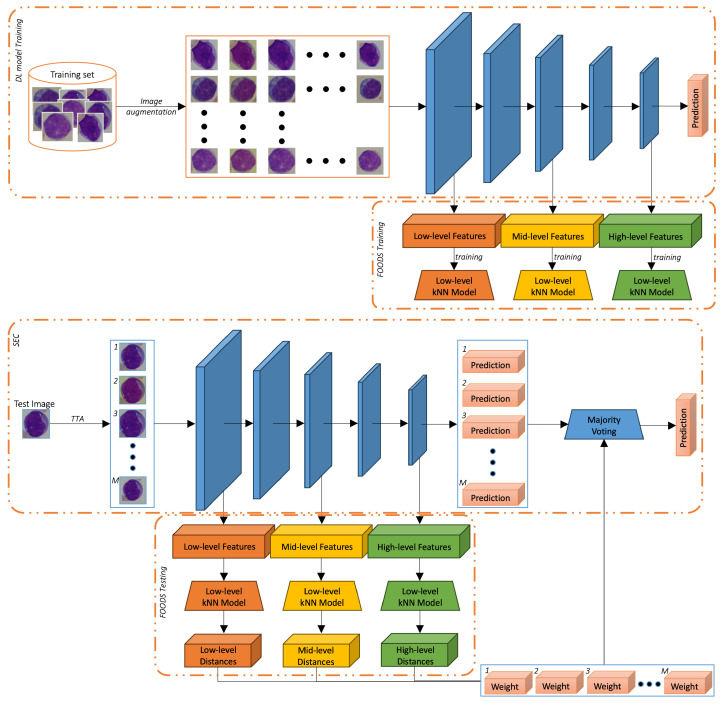
Proposed method. (**Top**): the training step, involving the training of the DL model with the set of augmentations *A* and the FOODS initialisation. (**Bottom**): the testing step, including TTA with *A*, image filtering with FOODS, and final prediction with SEC.

Our methodology is composed of three main components: the TTA procedure, the filtering of OOD samples (FOODS) procedure, and a Self-Ensemble with Confidence (SEC) classifier. In Figure 1, we reported the whole pipeline of our methodology. To ease readability, we reported separately the training and the testing steps. The TTA procedure mainly aims to generate alternative versions of the original target images that can be used to improve the image representation by fusing different sources of information. Given that *A* has already been used during training of the DL model *C* to produce alternative images of the source dataset (as reported in the top image of Figure 1) and given that such alternative images lie within the domain of the class, *A* can also be used during testing to create alternative images of the target dataset as outlined in Algorithm 1.
**Algorithm 1:** TTA    **Require:** a set of augmentations *A* and an input image *I*    **Ensure:**
*M* = |*A*| transformed images      1: *M*_0_ = *I*      2: **for**
*i* = 1, …, |*A*| **do**      3:  compute *M_i_* = *A_i_(I)*      4: **end for**

However, as mentioned before, we cannot assume that the augmented images generated from the target set will always lie within the distribution of the known classes. This assumption is particularly fragile in cross-domain scenarios, in particular in the medical field, where data acquisition protocols, device characteristics, or patient populations may vary significantly across institutions. As a result, TTA may lead to unrealistic or OOD samples that are too far from the original sample distribution and could negatively impact the final prediction. FOODS aims at removing those OOD samples by searching for images that are significantly dissimilar to the original training data. For this purpose, we used a search strategy based on k-Nearest Neighbours (kNN) with Euclidean distance, which avoids the storage and inversion of large covariance matrices (as in Mahalanobis distance) while still providing a robust unsupervised OOD filtering mechanism based on local density estimation in the deep feature space. To improve the robustness of the filtering operation, we exploited multi-layer deep features. The overall procedure is summarised below. FOODS initialisation comprises the following:

- Multi-Layer Feature Extraction: For all training images, features are extracted from three layers of a neural network, namely “low”, “mid”, and “high” levels (the precise layer depends on the used architecture).
- kNN Model Training: For each selected feature layer (“low”, “mid”, “high”), a separate kNN model is trained. This creates a non-parametric model of the feature distribution.
- Distance Threshold Estimation: For each training image, the average distance to its k nearest neighbours (e.g., k = 5) is computed. The 95th percentile of these distances is used as a threshold to distinguish ID from OOD samples. This choice is in line with standard practices in outlier detection, where even training samples lying beyond a high percentile (such as the 95th) are regarded as outliers. Such a conservative filtering ensures that only TTA images with features sufficiently close to the training data distribution are retained.

At testing time, the following occur:

- TTA Feature Extraction: Each TTA image is processed to extract features from the three layers.
- TTA Distance Calculation: For each TTA feature vector, its average kNN distance is computed using the corresponding trained kNN model.
- TTA Image Filtering: A TTA image is retained only if its average distance is below the above-mentioned threshold for all considered layers. Filtering an image in practice corresponds to assigning it a weight of 0 in the subsequent SEC fusion step.
- TTA Weighting: For all retained TTA images, a weight is assigned that is inversely proportional to their average distance, giving more importance to augmentations that are closer to the training distribution.

Finally, SEC aims at enhancing the prediction robustness by merging the original and the TTA-filtered samples into a single prediction similar to a previous DG work [51], where features extracted from augmented images were then aggregated to create a more robust feature descriptor in Person Re-Identification addressed, as usual, as an image retrieval problem [51]. This work, instead, focuses on image classification and exploits a Self-Ensemble (SE) classifier. SEs are a special case of ensembles that, unlike common ensemble approaches, use a single DL model at inference time, reducing the costs and memory consumption during inference. Nevertheless, existing SEs are still created by fusing several DL models trained on different portions of a dataset or on different datasets [52], by fusing different checkpoints obtained in the same training process [53], or by injecting random noise at different layers [54]. Our proposed SEC, instead, introduces a more lightweight and practical alternative that avoids retraining or maintaining multiple models. It leverages, at test time, the knowledge already learned by a DL model *C* that was trained using original images and their augmented versions generated through a transformation function *A*. As a result, our method introduces a runtime overhead proportional to the number of augmentations (≈15× in our setup), while the memory footprint remains essentially unchanged, as FOODS and SEC operate sequentially without requiring storage of large intermediate features.

SEC adopts a late fusion strategy by aggregating the predictions of the original test image and its retained TTA samples. The final output is computed using a weighted soft voting mechanism [14], where each sample’s contribution is determined by its similarity to the training distribution as estimated by FOODS. We outline this very simple and parameter-free procedure as pseudo-code in Algorithm 2.
**Algorithm 2:** SEC    **Require:** a set *M* of different versions of the original image *I*, the corresponding          weights *w*, and a DL model *C*    **Ensure:** a prediction *p* for the image *I*      1: initialise *p* = 0      2: **for**
*i* = 0, …, |*M*| **do**      3:  compute *p* = *p* + *C*(*M_i_*) ∗ *w_i_*      4: **end for**      5: *p* = *p*/|*M*|      6: *p* = *argmax*(*p*)

It must also be noted that the proposed method is compatible with any DL architecture since it is not based on any specific DL architecture and thus can be considered model-agnostic.

## 4. Experimental Evaluation

This section overviews the materials (Section 4.1), methods (Section 4.2), and experimental setup (Section 4.3). Finally, Section 4.4 presents the obtained results.

### 4.1. Datasets

In our study, we selected four well-established and publicly available datasets to ensure the integrity and generalisability of our results across diverse data compositions. Even if several datasets for WBC sub-type classification exist within the literature, they have limitations in terms of image count for each WBC sub-type (around 50 images per class) [55] or lack representation of one class [56], further limiting their applicability in our investigation. To circumvent these constraints and foster a comprehensive evaluation framework, we selected datasets encompassing a broader spectrum of WBC classes with substantial image counts. Subsequently, we eliminated or grouped the classes in advance according to the classes pertinent to our study. Such a selection strategy aims to mitigate biases stemming from dataset composition and to facilitate a robust comparison among the performance of different classification approaches.

The **Raabin-WBC** (from now on Raabin) dataset is a collection of 1145 images, with a size of 575×575 pixels, from different laboratories in Iran. The five WBC sub-types are represented as follows: lymphocytes (242), monocytes (242), neutrophils (242), eosinophils (201), and basophils (218) [57].

The **AML-Cytomorphology-LMU** (from now on AML) dataset presents a comprehensive collection of 18,365 expert-identified single-cell images with dimensions 400×400 categorised into 15 distinct classes [15]. For this study, we focused on the five main WBC classes. To this end, we grouped 8484 segmented neutrophils and 109 band neutrophils under a unified neutrophil class and merged 3937 typical lymphocytes with 11 atypical lymphocytes into a single lymphocyte class. The resulting class distribution is as follows: neutrophils (8593), lymphocytes (3948), monocytes (1789), eosinophils (424), and basophils (79).

The **PBC** dataset comprises 17,092 single-cell images sized 360×363 pixels collected at the Core Laboratory of Hospital Clinic of Barcelona from 2015 to 2019 and categorised into eight groups. For the purpose of this study, we considered again the five main WBC classes: neutrophils (3329), eosinophils (3117), basophils (1218), lymphocytes (1214), and monocytes (1420) [20,58].

**Figure 2:**
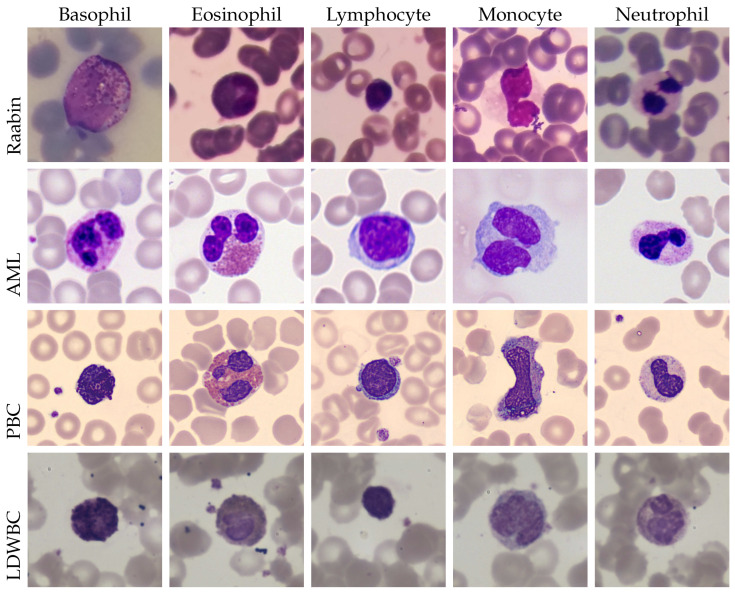
A comprehensive overview of the four datasets utilised in this study: Raabin, AML, PBC, and LDWBC. The figure delineates the five adopted classes: basophil, eosinophil, lymphocyte, monocyte, and neutrophil.

The **LDWBC** dataset consists of 22,645 images, with a size of 1280×1280 pixels, and originates from 150 microscope slides belonging to 150 healthy individuals [59]. The five WBC sub-types are represented as follows: 10,445 lymphocytes, 10,469 neutrophils, 968 monocytes, 539 eosinophils, and 224 basophils. A sample image for each dataset and each WBC sub-type is shown in Figure 2.

### 4.2. Deep Learning Architectures

To ensure the robustness of our findings against network-specific nuances, we evaluated diverse DL architectures by selecting one representative architecture from each category: linear networks, residual networks, lightweight networks, densely connected networks, inception networks, vision transformers, and hierarchical vision transformers. Among them, the chosen architectures were VGG-19 [60], ResNet-152 (from now on Res.152) [61], MobileNetV3 (from now on Mob.v3) [62], DenseNet-121 (from now on Den.121) [63], InceptionV3 (from now on Inc.v3) [64], ViT [65], and Swin [66]. Note that even if architectures specifically designed for WBC classification exist [24,27], we chose more general and well-known classification models in order to avoid different kinds of supervision or settings. All such DL architectures were first pretrained on the well-known ImageNet dataset [67] and then adapted following an established procedure [68], which consists of retaining all layers except the final fully connected layer, which is replaced with a new one to suit the specific target object categories (five in our study).

### 4.3. Experimental Setup

Cross-dataset experiments were performed, where each dataset was alternately designated as the source (training) dataset and the other was used as the target (testing) dataset. To ensure fair and direct comparisons on the same testing partition, all datasets were divided into three fixed subsets: training (60%), validation (20%), and testing (20%). Stratified sampling maintained the original data distribution. Due to class imbalances, a weighted random sampling technique was used during training to ensure equal representation of each class within each batch, coupled with a data augmentation procedure.

In this work, we have not investigated the optimal augmentation set for this task, as it is beyond the scope of our study. For this reason, we used standard augmentations since any augmentation set could be employed during training. We used a set of |A|=14 augmentations, including 7 centre croppings with strides ranging from 5 to 20, horizontal flipping, vertical flipping, rotation with strides in 5, 10, and 20, Gaussian blurring, and colour jitter. During testing, the TTA procedure, as explained in Algorithm 1, was executed with the same set of transformations mentioned before. It is important to note that during testing, these transformations were applied deterministically to ensure reproducibility and facilitate comparison in our experiments.

All experiments were carried out on a single machine equipped with an Intel(R) Xeon(R) CPU E5-2670 v3 @ 2.30 GHz, 128 GB RAM, and an NVIDIA Quadro GPU with 24 GB VRAM. Models were trained for a maximum of 100 epochs, with early stopping based on validation performance. We used a batch size of 32, a Stochastic Gradient Descent (SGD) optimiser, and a fixed learning rate of 0.001 throughout all training procedures.

We set the number of neighbours k=5 in the FOODS procedure to balance robustness and sensitivity. This choice is commonly adopted in OOD detection and filtering tasks as it provides a stable local estimate of the feature distribution while avoiding overfitting to single outliers or noise in the feature space. Additionally, using a small odd value like 5 ensures consistency in majority-based decisions while keeping the computational cost low. Preliminary experiments with other values (e.g., k=3 and k=7) yielded similar trends but showed slightly more variance in the filtering outcomes, confirming k=5 as a reliable trade-off between precision and generalisation. Furthermore, when submitted to the Wilcoxon signed-rank test, the differences across these *k* settings were not statistically significant, highlighting that extrapolating definitive conclusions from such closely aligned results is inherently difficult.

### 4.4. Experimental Results

The cross-dataset results obtained using the proposed TTA + FOODS + SEC method on the aforementioned benchmark datasets, across the considered DL architectures, are presented in Table 1, Table 2, Table 3 and Table 4 respectively. For each setting, we report accuracy (A), precision (P), Recall (R), and F1-score (F1). These metrics are computed on a per-class basis and subsequently aggregated using a weighted average to obtain a consolidated performance measure that accounts for class imbalances. For comparison, we also include results from the Baseline, representing standard inference without any test-time augmentation, as well as the performance of TTA+SEC without the FOODS filtering step. This latter configuration can be interpreted as a preliminary ablation study, highlighting the specific contribution of the FOODS module to the overall performance.

Despite its simplicity, the proposed solution often leads to tangible improvements, particularly when Raabin is used as the target dataset, where performance gains are consistently observed across all tested DL architectures. This may be attributed to the fact that Raabin was collected across multiple hospitals, introducing a higher degree of variability than the other datasets. Overall, especially when compared to traditional TTA, the results confirm that the proposed approach can enhance model performance.

When LDWBC was employed as the target dataset, instead, the improvements brought by our method were, in some cases, marginal and, for certain architectures, even slightly negative. We attribute this behaviour mainly to the intrinsic properties of LDWBC, which is composed of uniformly acquired, high-resolution images from a single laboratory, leading to a more homogeneous distribution and generally strong Baseline performance. Under such conditions of low acquisition variability, the added diversity generated by test-time augmentations may offer limited benefits and, in some cases, introduce mild perturbations that FOODS cannot fully compensate for. This aspect highlights a natural limitation of augmentation-based test-time strategies: they are especially effective under strong domain shifts (as in Raabin or AML), whereas their contribution is less pronounced when the target distribution is already highly consistent.

However, the main limitation of our method lies in the inherent randomness introduced by TTA in the feature space. Given the assumption that no prior knowledge of the target dataset is available, it becomes infeasible to compute meaningful statistics to determine which specific set of augmentations would most effectively transform the feature space for a given target dataset and DL architecture. As a result, improvements are not always consistent across all evaluation metrics and, in some cases, may appear marginal or statistically insignificant.

In addition, in this work, we deliberately adopted a fixed set of standard augmentations to ensure reproducibility, fairness across datasets, and comparability among architectures. Beyond reproducibility, the choice of simple geometric (flips, rotations, crops) and mild photometric transformations was motivated by their coherence with the typical morphology and aspect of leukocytes, which present relatively stable circular/elliptical shapes and consistent staining patterns across laboratories. Such operations simulate realistic variability in microscopy—e.g., cell orientation, slight misalignment, or small illumination shifts—without altering essential biological structures.

To assess the statistical significance of the observed improvements, we conducted a Wilcoxon signed-rank test, comparing the Baseline with the proposed method. Additionally, this test served as a complementary ablation study to evaluate the contribution of the SEC module, which, as previously described, employs a weighted soft voting strategy. Specifically, we alternately removed the FOODS component, the use of learned weights for voting, and replaced the soft voting mechanism with a hard voting approach to evaluate the individual contributions of each element. Note that, in some ablation settings, we used the weights derived from FOODS to modulate the voting process without performing any actual OOD filtering. In these cases, all TTA samples were retained regardless of their distance from the training distribution, but their contribution to the final prediction was scaled based on their similarity to the training data, as estimated by the kNN distance. To facilitate result visualisation and comparison, a single statistical test was performed by aggregating the scores across all datasets and DL architectures. Accordingly, in Table 5, for each tested configuration, we report the statistical difference (i.e., the average performance gap with respect to the Baseline) and the *p*-value obtained from the Wilcoxon signed-rank test.

As shown in in Table 5, the use of soft voting over hard voting provides a substantial contribution both in terms of performance differences and statistical significance, highlighting the importance of leveraging confidence-aware fusion strategies. The final configuration, which integrates all the components of the proposed method (FOODS-based filtering, distance-based weighting, and soft voting), consistently yields the most significant performance gains, confirming the effectiveness of each individual module and their synergistic combination.

**Figure 3:**
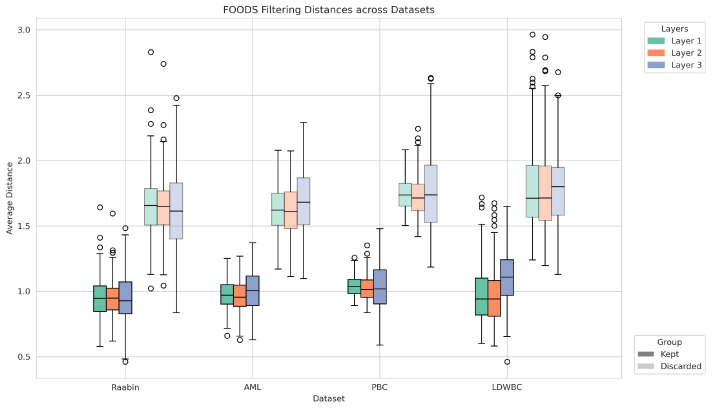
Distribution of normalised distances (using the threshold computed during training) in the deep feature space for augmented samples across datasets and layers. Boxplots are grouped by dataset, with colours indicating feature layers and solid colours indicating kept images and transparent colours indicating discarded images.

To provide a more intuitive understanding of the effect of FOODS, we report in Figure 3 the distribution of distances in the deep feature space for augmented samples that were retained (Kept) or discarded (Discarded). To ensure comparability across different layers and to improve visualisation, distances were normalised by the threshold value estimated during training. As expected, retained samples exhibit consistently lower normalised distances, whereas discarded samples show higher deviations, confirming that FOODS effectively filters out unreliable augmentations.

Overall, the experimental results confirm that the proposed TTA + FOODS + SEC pipeline is a robust and effective strategy for enhancing prediction performance in cross-domain scenarios, especially in the medical imaging context. The method improves model reliability without requiring retraining or architectural modifications by filtering out potentially harmful augmentations and adaptively weighting informative ones. This makes it a practical and generalizable solution for domain-shifted inference tasks.

## 5. Conclusions

In this work, we presented a novel test-time inference pipeline combining test-time augmentation (TTA), an Out-of-Distribution filtering strategy (FOODS), and a Self-Ensembling mechanism with Confidence-based voting (SEC). The proposed method aims to improve the robustness and accuracy of deep learning models in cross-domain scenarios, particularly in medical imaging, where domain shifts are common and challenging. Moreover, the proposed solution is model-agnostic, does not require model retraining, and introduces a negligible computational overhead at inference time. In terms of computational resources, our method introduces a runtime overhead proportional to the number of augmentations (≈15× in our setup), while the memory footprint remains essentially unchanged, as FOODS and SEC operate sequentially without requiring storage of large intermediate features. This makes it suitable for practical deployment, even in resource-constrained environments.

Our extensive cross-dataset experiments demonstrated that the proposed pipeline consistently improves predictive performance across multiple architectures and datasets. Notably, the FOODS module was effective in filtering unrealistic augmented samples, while the SEC module leveraged the retained samples via a weighted soft voting, contributing to more reliable predictions.

Despite its promising results, our method relies on the assumption that the training set sufficiently represents the In-Distribution space. Additionally, the use of a fixed and relatively small set of augmentations (|A|=14) may be seen as a limitation. However, this choice was made intentionally to simplify computation, ensure reproducibility, and enable fair comparisons across different settings. In addition, stronger or more diverse augmentations may increase the likelihood of producing OOD samples, making filtering more critical. Conversely, milder augmentations may reduce the filtering effect, limiting potential gains. In practice, the method could be extended to leverage a broader set of augmentations, as the FOODS component has proven effective in adaptively filtering out OOD samples and prioritising those transformations that yield features more aligned with the training distribution.

Future research directions could include investigating alternative ways to overcome the previously mentioned limitations, combining our solution with other paradigms and methods to address domain shift, and assessing the effectiveness of our solution in further medical (and non-medical) applications affected by restrictive privacy-preserving policies like the one addressed in this work. Finally, since we restricted ourselves to a simple set of geometric and mild photometric augmentations, as these remain coherent with the typical circular/elliptical shapes and staining aspects of leukocytes, future studies will explore in detail how individual transformations may differently help or harm under domain shift.

## Figures and Tables

**Table 1:** Cross-dataset performances of the proposed TTA+ method, compared with the traditional inference approach (Baseline) and the TTA without FLOODs on the Raabin dataset.

Arc.	Method	AML ⟶ Raabin				PBC ⟶ Raabin				LDWBC ⟶ Raabin			
		**A**	**P**	**R**	**F1**	**A**	**P**	**R**	**F1**	**A**	**P**	**R**	**F1**
VGG-19	Baseline	54.1	62.8	54.1	48.4	65.5	72.0	65.5	58.9	51.5	55.5	51.5	45.6
	TTA	55.9	63.4	55.9	50.5	67.2	73.2	67.2	61.6	49.8	53.8	49.8	43.4
	TTA+	55.9	63.4	55.9	50.5	67.2	73.6	67.2	61.7	49.8	53.9	49.8	43.4
Res.152	Baseline	64.2	79.9	64.2	56.4	63.3	67.8	63.3	60.2	44.5	43.5	44.5	36.0
	TTA	65.1	79.8	65.1	57.8	62.9	67.4	62.9	59.5	46.3	50.8	46.3	38.2
	TTA+	65.9	80.0	65.9	58.6	63.8	68.5	63.8	60.5	46.3	50.8	46.3	38.4
Inc.v3	Baseline	62.9	73.7	62.9	59.3	65.5	74.5	65.5	62.8	45.0	51.0	45.0	37.6
	TTA	63.3	73.7	63.3	60.7	67.7	79.5	67.7	64.9	45.9	50.7	45.9	38.6
	TTA+	64.2	74.0	64.2	62.1	68.6	80.2	68.6	65.7	45.9	50.7	45.9	38.6
Den.121	Baseline	40.2	60.4	40.2	31.9	63.8	67.6	63.8	60.5	51.5	62.0	51.5	45.3
	TTA	41.5	61.0	41.5	33.3	62.9	67.5	62.9	58.6	50.7	59.0	50.7	43.8
	TTA+	41.9	61.8	41.9	33.7	62.9	67.5	62.9	58.6	51.1	59.7	51.1	44.7
Mob.v3	Baseline	53.7	58.4	53.7	50.9	47.6	38.9	47.6	38.7	32.3	47.7	32.3	31.8
	TTA	57.6	63.6	57.6	55.9	53.3	48.1	53.3	44.0	33.6	48.9	33.6	32.8
	TTA+	60.3	65.6	60.3	58.7	55.9	55.3	55.9	46.6	33.6	48.9	33.6	32.8
ViT	Baseline	65.5	67.0	65.5	61.6	68.1	74.0	68.1	63.9	59.8	63.6	59.8	57.9
	TTA	67.2	68.4	67.2	64.2	70.3	76.7	70.3	66.4	64.6	68.7	64.6	63.8
	TTA+	67.2	69.0	67.2	64.9	71.2	77.2	71.2	67.6	66.4	69.0	66.4	65.5
Swin	Baseline	63.3	66.4	63.3	55.6	67.7	77.1	67.7	61.5	71.2	74.2	71.2	69.6
	TTA	63.8	65.1	63.8	57.0	69.0	73.9	69.0	63.8	71.6	75.1	71.6	69.7
	TTA+	63.8	66.5	63.8	57.1	69.4	76.7	69.4	64.3	71.6	75.1	71.6	69.9

**Table 2:** Cross-dataset performances of the proposed TTA+ method, compared with the traditional inference approach (Baseline) and the TTA without FLOODs on the AML dataset.

Arc.	Method	Raabin ⟶ AML				PBC ⟶ AML				LDWBC ⟶ AML			
		**A**	**P**	**R**	**F1**	**A**	**P**	**R**	**F1**	**A**	**P**	**R**	**F1**
VGG-19	Baseline	63.4	70.7	63.4	64.6	83.5	84.0	83.5	83.6	78.9	80.3	78.9	77.4
	TTA	63.9	72.2	63.9	65.6	83.5	84.5	83.5	83.8	79.9	83.0	79.9	78.5
	TTA+	65.5	73.0	65.5	66.7	86.1	86.6	86.1	86.1	80.4	83.4	80.4	78.9
Res.152	Baseline	76.8	77.7	76.8	77.1	73.2	80.5	73.2	72.9	66.5	78.7	66.5	62.8
	TTA	77.3	78.2	77.3	77.5	73.2	81.4	73.2	72.9	68.6	77.8	68.6	64.7
	TTA+	77.3	78.2	77.3	77.5	73.2	81.4	73.2	72.9	69.1	78.0	69.1	65.5
Inc.v3	Baseline	71.1	77.7	71.1	73.3	77.8	81.3	77.8	76.7	67.5	75.6	67.5	65.5
	TTA	73.2	79.4	73.2	75.0	74.2	79.9	74.2	73.3	73.2	77.8	73.2	71.5
	TTA+	74.2	80.1	74.2	76.0	74.2	80.1	74.2	73.3	73.2	78.1	73.2	71.6
Den.121	Baseline	77.8	77.7	77.8	76.0	74.7	80.7	74.7	74.4	78.4	82.4	78.4	75.8
	TTA	79.4	79.7	79.4	77.5	73.2	80.3	73.2	72.8	79.4	82.9	79.4	76.8
	TTA+	79.9	80.0	79.9	78.0	74.2	80.9	74.2	73.8	80.4	83.7	80.4	77.9
Mob.v3	Baseline	44.3	65.6	44.3	43.9	69.1	73.9	69.1	66.6	52.1	47.5	52.1	46.3
	TTA	51.5	72.3	51.5	53.3	70.1	75.0	70.1	68.1	54.1	46.7	54.1	47.9
	TTA+	52.1	73.1	52.1	53.9	71.6	76.2	71.6	69.8	54.6	47.2	54.6	48.3
ViT	Baseline	56.7	67.1	56.7	56.7	64.9	79.0	64.9	65.2	58.8	70.0	58.8	56.0
	TTA	58.8	69.8	58.8	59.4	67.5	79.0	67.5	66.5	59.8	70.0	59.8	57.9
	TTA+	59.3	70.1	59.3	60.1	67.5	79.0	67.5	66.5	61.9	71.3	61.9	60.3
Swin	Baseline	62.9	70.2	62.9	63.3	69.6	75.8	69.6	67.5	58.8	71.9	58.8	51.0
	TTA	61.3	71.7	61.3	62.6	72.7	78.1	72.7	71.7	59.8	72.6	59.8	52.7
	TTA+	63.9	73.2	63.9	64.9	72.7	78.1	72.7	71.7	60.3	73.0	60.3	53.1

**Table 3:** Cross-dataset performances of the proposed TTA+ method, compared with the traditional inference approach (Baseline) and the TTA without FLOODs on the PBC dataset.

Arc.	Method	Raabin ⟶ PBC				AML ⟶ PBC				LDWBC ⟶ PBC			
		**A**	**P**	**R**	**F1**	**A**	**P**	**R**	**F1**	**A**	**P**	**R**	**F1**
VGG-19	Baseline	57.9	55.7	57.9	53.1	42.6	57.5	42.6	38.1	61.2	68.1	61.2	56.9
	TTA	62.4	61.9	62.4	58.0	49.6	62.9	49.6	45.6	69.4	74.6	69.4	67.6
	TTA+	62.4	61.9	62.4	58.0	51.2	64.5	51.2	48.0	75.2	78.8	75.2	74.0
Res.152	Baseline	40.1	50.6	40.1	32.4	43.8	70.3	43.8	34.5	52.5	54.0	52.5	44.2
	TTA	43.0	50.2	43.0	35.3	48.3	74.1	48.3	39.0	59.9	56.8	59.9	52.9
	TTA+	44.2	52.4	44.2	36.9	49.6	74.9	49.6	40.9	62.0	77.8	62.0	55.5
Inc.v3	Baseline	47.5	47.5	47.5	39.5	40.9	68.2	40.9	32.3	25.2	15.8	25.2	14.1
	TTA	51.2	49.4	51.2	42.5	40.9	58.6	40.9	32.9	32.6	40.3	32.6	22.0
	TTA+	52.1	69.1	52.1	43.4	44.2	61.2	44.2	38.6	37.6	59.9	37.6	27.0
Den.121	Baseline	41.3	55.6	41.3	34.2	27.3	11.0	27.3	15.5	82.2	85.7	82.2	81.2
	TTA	45.5	58.6	45.5	38.9	31.4	12.8	31.4	18.2	88.0	89.8	88.0	87.6
	TTA+	46.3	58.9	46.3	40.0	32.6	13.4	32.6	19.0	89.7	91.3	89.7	89.4
Mob.v3	Baseline	55.0	55.6	55.0	48.4	71.1	78.7	71.1	70.1	33.5	47.8	33.5	25.4
	TTA	56.6	58.0	56.6	50.6	79.8	84.2	79.8	79.6	36.8	48.4	36.8	28.0
	TTA+	59.5	58.7	59.5	53.6	81.4	84.9	81.4	81.3	39.7	49.5	39.7	31.2
ViT	Baseline	59.9	58.4	59.9	54.5	79.3	83.4	79.3	78.9	63.6	77.7	63.6	61.7
	TTA	62.0	63.9	62.0	57.0	83.5	86.3	83.5	83.4	65.3	78.0	65.3	64.0
	TTA+	62.0	63.9	62.0	57.0	85.5	88.0	85.5	85.4	67.4	78.6	67.4	66.3
Swin	Baseline	60.3	61.8	60.3	54.2	52.9	72.9	52.9	43.4	71.9	82.6	71.9	71.4
	TTA	60.7	60.6	60.7	54.7	56.2	73.4	56.2	48.9	76.4	83.6	76.4	75.9
	TTA+	61.2	62.7	61.2	55.8	58.3	74.7	58.3	51.3	78.1	84.7	78.1	77.7

**Table 4:** Cross-dataset performances of the proposed TTA+ method, compared with the traditional inference approach (Baseline) and the TTA without FLOODs on the LDWBC dataset.

Arc.	Method	Raabin ⟶ LDWBC				AML ⟶ LDWBC				PBC ⟶ LDWBC			
		**A**	**P**	**R**	**F1**	**A**	**P**	**R**	**F1**	**A**	**P**	**R**	**F1**
VGG-19	Baseline	58.2	65.3	58.2	59.7	42.7	39.7	42.7	38.5	67.6	68.7	67.6	64.6
	TTA	54.7	64.4	54.7	56.3	41.8	39.7	41.8	37.7	66.7	67.1	66.7	63.6
	TTA+	55.1	65.3	55.1	56.7	42.2	41.1	42.2	38.0	67.1	67.6	67.1	64.1
Res.152	Baseline	53.3	55.5	53.3	49.0	50.7	52.3	50.7	45.8	79.1	81.1	79.1	78.2
	TTA	54.2	58.5	54.2	50.8	50.7	52.1	50.7	45.4	78.7	81.2	78.7	77.7
	TTA+	54.7	59.8	54.7	51.6	50.7	53.3	50.7	45.4	78.7	81.2	78.7	77.7
Inc.v3	Baseline	51.6	60.4	51.6	52.1	45.8	50.7	45.8	41.8	59.1	61.7	59.1	59.0
	TTA	52.0	61.8	52.0	52.0	41.8	46.9	41.8	38.4	61.8	62.7	61.8	60.7
	TTA+	54.2	63.0	54.2	54.1	42.7	47.6	42.7	41.6	62.2	66.9	62.2	60.9
Den.121	Baseline	45.3	62.6	45.3	41.0	40.9	38.2	40.9	33.0	61.3	62.2	61.3	59.4
	TTA	45.8	54.3	45.8	40.6	39.6	33.9	39.6	31.6	65.8	69.4	65.8	63.3
	TTA+	45.8	54.4	45.8	41.0	39.6	35.8	39.6	31.6	65.8	69.4	65.8	63.3
Mob.v3	Baseline	47.6	62.1	47.6	46.3	39.1	50.6	39.1	33.0	57.8	71.9	57.8	52.8
	TTA	50.2	64.8	50.2	49.9	39.1	50.1	39.1	32.6	57.8	66.0	57.8	52.4
	TTA+	50.2	65.2	50.2	50.0	40.4	52.0	40.4	34.5	59.1	67.4	59.1	54.1
ViT	Baseline	55.1	66.3	55.1	53.3	62.7	75.1	62.7	61.9	66.2	69.9	66.2	65.2
	TTA	52.9	68.2	52.9	50.4	65.3	76.8	65.3	64.8	72.0	74.3	72.0	71.5
	TTA+	53.3	70.6	53.3	51.0	68.0	77.1	68.0	67.4	72.0	74.7	72.0	71.5
Swin	Baseline	52.9	56.5	52.9	50.2	44.9	40.5	44.9	35.8	60.4	71.2	60.4	57.0
	TTA	52.0	58.3	52.0	49.6	46.2	55.7	46.2	39.2	61.8	72.0	61.8	58.5
	TTA+	52.0	58.4	52.0	49.6	46.2	60.4	46.2	39.2	61.8	72.2	61.8	58.6

**Table 5:** Statistical comparison of different ablation settings using the Wilcoxon signed-rank test. Each configuration is defined by the presence (✓) or absence (✗) of the components TTA and FOODS, and the type of weights and voting strategy. The reported values represent the average performance difference (Diff.) with respect to the Baseline and the corresponding *p*-value, computed by aggregating results across all datasets and DL architectures.

		SEC		Stats	
TTA	FOODS	Voting	Weights	Diff.	*p*-Value
✓	✗	Hard	Uniform	0.893	0.0547
✓	✗	Hard	Weighted	0.784	0.0838
✓	✗	Soft	Uniform	1.234	0.0499
✓	✗	Soft	Weighted	1.235	0.0212
✓	✓	Hard	Uniform	1.009	0.0455
✓	✓	Hard	Weighted	0.978	0.0429
✓	✓	Soft	Uniform	1.267	0.0104
✓	✓	Soft	Weighted	1.354	0.0102

## Data Availability

The original data presented in the study are openly available in the Raabin Health Database at https://raabindata.com/, in the PBC dataset at https://github.com/paperswithcode/paperswithcode-data, in the AML dataset at https://www.cancerimagingarchive.net/collection/aml-cytomorphology\_lmu/, and in the LDWBC dataset at http://ldwbc.biodwhu.cn/LDWBC/ (accessed on 24 July 2025).

## Abbreviations

The following abbreviations are used in this manuscript:

AI	Artificial Intelligence
CNN	Convolutional Neural Network
DA	domain adaptation
DG	domain generalisation
DL	Deep Learning
DSEB	depthwise squeeze-and-excitation block
FOODS	filtering of OOD samples
ID	In-Distribution
k-NN	k-Nearest Neighbour
ML	Machine Learning
OOD	Out-Of-Distribution
PPM	pyramid pooling module
SE	Self-Ensemble
SEC	Self-Ensemble with Confidence
SGD	Stochastic Gradient Descent
TTA	test-time augmentation
UDA	Unsupervised Domain Adaptation
ViT	vision transformer
WBC	white blood cell
WBCC	Whole Blood Cell Count

## References

[1] Burton A.G., Jandrey K.E. (2018). Leukocytosis and Leukopenia. Textbook of Small Animal Emergency Medicine.

[2] Kutlu H., Avci E., Özyurt F. (2020). White blood cells detection and classification based on regional convolutional neural networks. Med. Hypotheses.

[3] Pandey P., P P.A., Kyatham V., Mishra D., Dastidar T.R. (2020). Target-Independent Domain Adaptation for WBC Classification Using Generative Latent Search. IEEE Trans. Med. Imaging.

[4] Salehi R., Sadafi A., Gruber A., Lienemann P., Navab N., Albarqouni S., Marr C. (2022). Unsupervised Cross-Domain Feature Extraction for Single Blood Cell Image Classification. Proceedings of the Medical Image Computing and Computer Assisted Intervention—MICCAI.

[5] Wang J., Lan C., Liu C., Ouyang Y., Qin T., Lu W., Chen Y., Zeng W., Yu P.S. (2023). Generalizing to Unseen Domains: A Survey on Domain Generalization. IEEE Trans. Knowl. Data Eng..

[6] Lim S., Kim I., Kim T., Kim C., Kim S. (2019). Fast AutoAugment. In Neural Information Processing Systems—NeurIPS. https://proceedings.neurips.cc/paper_files/paper/2019/file/6add07cf50424b14fdf649da87843d01-Paper.pdf.

[7] Muller S.G., Hutter F. (2021). TrivialAugment: Tuning-free Yet State-of-the-Art Data Augmentation. Proceedings of the International Conference on Computer Vision—ICCV.

[8] Kimura M. (2021). Understanding Test-Time Augmentation. Lecture Notes in Computer Science (Including Subseries Lecture Notes in Artificial Intelligence and Lecture Notes in Bioinformatics).

[9] Ibrahim A.T., Abdullahi M., Kana A.F.D., Mohammed M.T., Hassan I.H. (2025). Categorical classification of skin cancer using a weighted ensemble of transfer learning with test time augmentation. Data Sci. Manag..

[10] Cino L., Distante C., Martella A., Mazzeo P.L. (2025). Skin Lesion Classification Through Test Time Augmentation and Explainable Artificial Intelligence. J. Imaging.

[11] Garta I.Y., Tai S.K., Chen R.C. (2024). Improved Detection of Multi-Class Bad Traffic Signs Using Ensemble and Test Time Augmentation Based on Yolov5 Models. Appl. Sci..

[12] Fu R., Han J., Sun Y., Wang S., Al-Absi M.A., Wang X., Sun H. (2025). Robust crop disease detection using multi-domain data augmentation and isolated test-time adaptation. Expert Syst. Appl..

[13] Cho Y., Kim Y., Yoon J., Hong S., Lee D. Feature Augmentation Based Test-Time Adaptation. Proceedings of the 2025 IEEE Winter Conference on Applications of Computer Vision, WACV.

[14] Boulahia S., Amamra A., Madi M., Daikh S. (2021). Early, intermediate and late fusion strategies for robust deep learning-based multimodal action recognition. Mach. Vis. Appl..

[15] Matek C., Schwarz S., Spiekermann K., Marr C. (2019). Human-level recognition of blast cells in acute myeloid leukaemia with convolutional neural networks. Nat. Mach. Intell..

[16] Acevedo A., Merino A., Boldú L., Molina A., Alférez S., Rodellar J. (2021). A new convolutional neural network predictive model for the automatic recognition of hypogranulated neutrophils in myelodysplastic syndromes. Comput. Biol. Med..

[17] Vogado L.H., Veras R.M., Araujo F.H., Silva R.R., Aires K.R. (2018). Leukemia diagnosis in blood slides using transfer learning in CNNs and SVM for classification. Eng. Appl. Artif. Intell..

[18] Huang Q., Li W., Zhang B., Li Q., Tao R., Lovell N.H. (2020). Blood Cell Classification Based on Hyperspectral Imaging with Modulated Gabor and CNN. J. Biomed. Health Inform..

[19] Loddo A., Putzu L. (2021). On the Effectiveness of Leukocytes Classification Methods in a Real Application Scenario. AI.

[20] Acevedo A., Alférez S., Merino A., Puigví L., Rodellar J. (2019). Recognition of peripheral blood cell images using convolutional neural networks. Comput. Methods Programs Biomed..

[21] Rastogi P., Khanna K., Singh V. (2022). LeuFeatx: Deep learning-based feature extractor for the diagnosis of acute leukemia from microscopic images of peripheral blood smear. Comput. Biol. Med..

[22] Fırat H. (2024). Classification of microscopic peripheral blood cell images using multibranch lightweight CNN-based model. Neural Comput. Appl..

[23] Tavakoli S., Ghaffari A., Kouzehkanan Z.M., Hosseini R. (2021). New segmentation and feature extraction algorithm for classification of white blood cells in peripheral smear images. Sci. Rep..

[24] Rubin R., Anzar S.M., Panthakkan A., Mansoor W. Transforming Healthcare: Raabin White Blood Cell Classification with Deep Vision Transformer. Proceedings of the International Conference on Signal Processing and Information Security—ICSPIS.

[25] Saleem S., Amin J., Sharif M., Mallah G.A., Kadry S., Gandomi A.H. (2022). Leukemia segmentation and classification: A comprehensive survey. Comput. Biol. Med..

[26] Das P.K., Meher S. (2021). An efficient deep Convolutional Neural Network based detection and classification of Acute Lymphoblastic Leukemia. Expert Syst. Appl..

[27] Long F., Peng J., Song W., Xia X., Sang J. (2021). BloodCaps: A capsule network based model for the multiclassification of human peripheral blood cells. Comput. Methods Programs Biomed..

[28] Tummala S., Suresh A.K. (2023). Few-shot learning using explainable Siamese twin network for the automated classification of blood cells. Med. Biol. Eng. Comput..

[29] Zhang R., Han X., Lei Z., Jiang C., Gul I., Hu Q., Zhai S., Liu H., Lian L., Liu Y. (2022). RCMNet: A deep learning model assists CAR-T therapy for leukemia. Comput. Biol. Med..

[30] Jiang L., Tang C., Zhou H. (2022). White blood cell classification via a discriminative region detection assisted feature aggregation network. Biomed. Opt. Express.

[31] Manzari O.N., Ahmadabadi H., Kashiani H., Shokouhi S.B., Ayatollahi A. (2023). MedViT: A robust vision transformer for generalized medical image classification. Comput. Biol. Med..

[32] Almalik F., Alkhunaizi N., Almakky I., Nandakumar K. (2023). FeSViBS: Federated Split Learning of Vision Transformer with Block Sampling. Proceedings of the Medical Image Computing and Computer Assisted Intervention—MICCAI.

[33] Bravin R., Nanni L., Loreggia A., Brahnam S., Paci M. (2023). Varied Image Data Augmentation Methods for Building Ensemble. IEEE Access.

[34] Li C., Liu Y. (2024). Improved Generalization of White Blood Cell Classification by Learnable Illumination Intensity Invariant Layer. IEEE Signal Process. Lett..

[35] Bairaboina S.S.R., Battula S.R. (2023). Ghost-ResNeXt: An Effective Deep Learning Based on Mature and Immature WBC Classification. Appl. Sci..

[36] Togaçar M., Ergen B., Cömert Z. (2020). Classification of white blood cells using deep features obtained from Convolutional Neural Network models based on the combination of feature selection methods. Appl. Soft Comput..

[37] Şengür A., Akbulut Y., Budak Ü., Cömert Z. (2019). White blood cell classification based on shape and deep features. Proceedings of the Artificial Intelligence and Data Processing Symposium—IDAP.

[38] Li C., Lin X., Mao Y., Lin W., Qi Q., Ding X., Huang Y., Liang D., Yu Y. (2022). Domain generalization on medical imaging classification using episodic training with task augmentation. Comput. Biol. Med..

[39] Roels J., Hennies J., Saeys Y., Philips W., Kreshuk A. (2019). Domain Adaptive Segmentation In Volume Electron Microscopy Imaging. Proceedings of the International Symposium on Biomedical Imaging—ISBI.

[40] Mahmood F., Chen R.J., Durr N.J. (2018). Unsupervised Reverse Domain Adaptation for Synthetic Medical Images via Adversarial Training. IEEE Trans. Med. Imaging.

[41] Miller T., Cheng J., Fu H., Gu Z., Xiao Y., Zhou K., Gao S., Zheng R., Liu J. (2020). Noise Adaptation Generative Adversarial Network for Medical Image Analysis. IEEE Trans. Med. Imaging.

[42] Li W., Yang D., Ma C., Liu L. (2023). Identifying novel disease categories through divergence optimization: An approach to prevent misdiagnosis in medical imaging. Comput. Biol. Med..

[43] Chen Z., Pan Y., Ye Y., Cui H., Xia Y. (2023). Treasure in Distribution: A Domain Randomization Based Multi-source Domain Generalization for 2D Medical Image Segmentation. Proceedings of the Medical Image Computing and Computer Assisted Intervention—MICCAI.

[44] Sadafi A., Salehi R., Gruber A., Boushehri S.S., Giehr P., Navab N., Marr C. (2023). A Continual Learning Approach for Cross-Domain White Blood Cell Classification. Proceedings of the Domain Adaptation and Representation Transfer—MICCAI Workshop.

[45] Umer R.M., Gruber A., Boushehri S.S., Metak C., Marr C. (2023). Imbalanced Domain Generalization for Robust Single Cell Classification in Hematological Cytomorphology. arXiv.

[46] Berson S.W. (2003). HIPAA. Oncol. Issues.

[47] Parera A.V., Costa X. (2018). General Data Protection Regulation. Data Protection Law in the EU: Roles, Responsibilities and Liability. https://gdpr-info.eu/.

[48] Li Q., Tan K., Yuan D., Liu Q. (2025). Progressive Domain Adaptation for Thermal Infrared Tracking. Electronics.

[49] Shu X., Huang F., Qiu Z., Zhang X., Yuan D. (2024). Learning Unsupervised Cross-Domain Model for TIR Target Tracking. Mathematics.

[50] Khosla A., Zhou T., Malisiewicz T., Efros A.A., Torralba A. Undoing the Damage of Dataset Bias. Proceedings of the European Conference on Computer Vision—ECCV.

[51] Putzu L., Loddo A., Delussu R., Fumera G. (2023). Specialise to Generalise: The Person Re-identification Case. Proceedings of the Image Analysis and Processing—ICIAP.

[52] Mitsuzum Y., Irie G., Kimura A., Nakazawa A. (2020). A Generative Self-Ensemble Approach To Simulated+Unsupervised Learning. Proceedings of the International Conference on Image Processing—ICIP.

[53] Ding H., Dai C., Wu Y., Ma W., Zhou H. (2024). SETEM: Self-ensemble training with Pre-trained Language Models for Entity Matching. Knowl.-Based Syst..

[54] Liu X., Cheng M., Zhang H., Hsieh C.J. Towards Robust Neural Networks via Random Self-ensemble. Proceedings of the European Conference on Computer Vision—ECCV.

[55] Rezatofighi S.H., Soltanian-Zadeh H. (2011). Automatic recognition of five types of white blood cells in peripheral blood. Comput. Med. Imaging Graph..

[56] Chen H., Liu J., Hua C., Zuo Z., Feng J., Pang B., Xiao D. TransMixNet: An Attention Based Double-Branch Model for White Blood Cell Classification and Its Training with the Fuzzified Training Data. Proceedings of the International Conference on Bioinformatics and Biomedicine—BIBM.

[57] Kouzehkanan Z.M., Saghari S., Tavakoli S., Rostami P., Abaszadeh M., Mirzadeh F., Satlsar E.S., Gheidishahran M., Gorgi F., Mohammadi S. (2022). A large dataset of white blood cells containing cell locations and types, along with segmented nuclei and cytoplasm. Sci. Rep..

[58] Acevedo A., Merino A., Alférez S., Molina Á., Boldú L., Rodellar J. (2020). A dataset of microscopic peripheral blood cell images for development of automatic recognition systems. Data Brief.

[59] Chen H., Liu J., Hua C., Feng J., Pang B., Cao D., Li C. (2022). Accurate classification of white blood cells by coupling pre-trained ResNet and DenseNet with SCAM mechanism. BMC Bioinform..

[60] Simonyan K., Zisserman A. Very Deep Convolutional Networks for Large-Scale Image Recognition. Proceedings of the International Conference on Learning Representations—ICLR.

[61] He K., Zhang X., Ren S., Sun J. (2016). Deep Residual Learning for Image Recognition. Proceedings of the Computer Vision and Pattern Recognition—CVPR.

[62] Howard A., Sandler M., Chen B., Wang W., Chen L.C., Tan M., Chu G., Vasudevan V., Zhu Y., Pang R. (2019). Searching for MobileNetV3. Proceedings of the International Conference on Computer Vision—ICCV.

[63] Huang G., Liu Z., van der Maaten L., Weinberger K.Q. (2017). Densely Connected Convolutional Networks. Proceedings of the Computer Vision and Pattern Recognition—CVPR.

[64] Szegedy C., Vanhoucke V., Ioffe S., Shlens J., Wojna Z. (2016). Rethinking the Inception Architecture for Computer Vision. Proceedings of the Computer Vision and Pattern Recognition—CVPR.

[65] Dosovitskiy A., Beyer L., Kolesnikov A., Weissenborn D., Zhai X., Unterthiner T., Dehghani M., Minderer M., Heigold G., Gelly S. An Image is Worth 16x16 Words: Transformers for Image Recognition at Scale. Proceedings of the International Conference on Learning Representations—ICLR.

[66] Liu Z., Lin Y., Cao Y., Hu H., Wei Y., Zhang Z., Lin S., Guo B. Swin Transformer: Hierarchical Vision Transformer using Shifted Windows. Proceedings of the International Conference on Computer Vision—ICCV.

[67] Deng J., Dong W., Socher R., Li L.J., Li K., Fei-Fei L. Imagenet: A large-scale hierarchical image database. Proceedings of the Computer Vision and Pattern Recognition—CVPR.

[68] Shin H., Roth H.R., Gao M., Lu L., Xu Z., Nogues I., Yao J., Mollura D.J., Summers R.M. (2016). Deep Convolutional Neural Networks for Computer-Aided Detection: CNN Architectures, Dataset Characteristics and Transfer Learning. IEEE Trans. Med. Imaging.

